# Supplementary material for: Effectiveness of an Educational Filmmaking Project in Promoting the Psychological Well-Being of Adolescents with Emotive/Behavioural Problems
Source: Healthcare (Basel). 2023 Jun 9;11(12):1695. doi: 10.3390/healthcare11121695 (PMC10297831; doi:10.3390/healthcare11121695)
Supplement: Supplementary file 1 [file healthcare-11-01695-s001.zip › healthcare-2352986-supplementary.pdf]

Supplementary Material

Supplementary File S1: All scales we used for testing our sample are reported in this supplementary section.

1. Rosenberg Self Esteem Scale (RSE);
2. Youth Self-Report scale (YSR);
3. Social Responsiveness Scale (SRS);
4. Revised Conners' Parent Rating Scale (CPRS-R).

Rosenberg Self-Esteem Scale

Scala dell'autostima di Rosenberg

(Copyright: Rosenberg, 1965)
(Versione Italiana: Prezza, Trombaccia, Armento, 1994)

---

1) Penso di valere almeno quanto gli altri

Fortemente d'accordo	D'accordo	In disaccordo	Fortemente in disaccordo
**3**	**2**	**1**	**0**

2) Penso di avere un certo numero di qualità

Fortemente d'accordo	D'accordo	In disaccordo	Fortemente in disaccordo
**3**	**2**	**1**	**0**

3) Sono portato a pensare di essere un vero fallimento

Fortemente d'accordo	D'accordo	In disaccordo	Fortemente in disaccordo
**0**	**1**	**2**	**3**

4) Sono in grado di fare le cose bene almeno come la maggior parte delle persone

Fortemente d'accordo	D'accordo	In disaccordo	Fortemente in disaccordo
**3**	**2**	**1**	**0**

5) Penso di non avere molto di cui essere fiero

Fortemente d'accordo	D'accordo	In disaccordo	Fortemente in disaccordo
**0**	**1**	**2**	**3**

6) Ho un atteggiamento positivo verso me stesso

Fortemente d'accordo	D'accordo	In disaccordo	Fortemente in disaccordo
**3**	**2**	**1**	**0**

7) Complessivamente sono soddisfatto di me stesso

Fortemente d'accordo	D'accordo	In disaccordo	Fortemente in disaccordo
**3**	**2**	**1**	**0**

8) Desidererei aver maggior rispetto di me stesso

Fortemente d'accordo	D'accordo	In disaccordo	Fortemente in disaccordo
**0**	**1**	**2**	**3**

9) Senza dubbio a volte mi sento inutile

Fortemente d'accordo	D'accordo	In disaccordo	Fortemente in disaccordo
**0**	**1**	**2**	**3**

10) A volte penso di essere un buono a nulla

Fortemente d'accordo	D'accordo	In disaccordo	Fortemente in disaccordo
**0**	**1**	**2**	**3**

Scala dell'autostima di Rosenberg - Risultati

Nome ________________________________ Cognome _________________________________ Età _________

Rater ___________________________________ Data esame ___________________ N: _____ / __________

Punteggio Totale ___________________

La scala va da 0 a 30. I punteggi tra 15 e 25 sono nel range di normalità; punteggi inferiori a 15 indicano una bassa autostima.

QUESTIONARIO SUL COMPORTAMENTO DEL GIOVANE -Anni 11-18
Youth Self Report for Ages 11-18 di Thomas M. Achenbach -2001 .

Per favore compila

INDICA IL TUO NOME E COGNOME			**PROFESSIONE DEI GENITORI**, **anche se attualmente non lavorano.** (Per favore specifica il tipo di professione-per esempio, meccanico, insegnante di scuola superiore, casalinga, operaio, tornitore, venditore di calzature, sergente dell'esercito.)<br><br>PADRE: Professione ____________<br>MADRE: Professione ____________
IL TUO SESSO:<br>☐M ☐F	LA TUA ETA':	GRUPPO ETNICO O RAZZA:	
DATA ODIERNA:		LA TUA DATA DI NASCITA:	
CLASSE FREQUENTATA ____________<br><br>NON FREQUENTO LA SCUOLA ☐		Se stai lavorando per favore indica il tipo di lavoro:	Per favore compila questo questionario che deve riflettere il tuo punto di vista anche se altre persone potrebbero non essere d'accordo. Sentiti libero di esprimere commenti aggiuntivi accanto ad ogni voce e nello spazio previsto a pag. 3 e 6. **Accertati di rispondere a tutte le domande.**

I. Per favore elenca gli sport che ami maggiormente praticare.
Per esempio: nuoto, baseball, pallacanestro, pattinaggio, ciclismo, pesca ecc.
☐ Nessuno

Rispetto agli altri ragazzi della tua età quanto tempo trascorri in ognuno degli sport elencati?

Rispetto agli altri ragazzi della tua età come pratichi ciascuno di questi sport?

	Meno degli altri	Come gli altri	Più degli altri	Meno degli altri	Come gli altri	Più degli altri
a.__________	☐	☐	☐	☐	☐	☐
b.__________	☐	☐	☐	☐	☐	☐
c.__________	☐	☐	☐	☐	☐	☐

II. Per favore elenca i tuoi hobby, attività, e giochi preferiti, oltre agli sport. Per esempio: carte, libri, pianoforte, macchine, computer, arte, etc. (Da non includere radio e TV)
☐ Nessuno

Rispetto agli altri ragazzi della tua età quanto tempo dedichi a ciascuna di queste attività?

Rispetto agli altri ragazzi della tua età quanto bene svolgi ciascuna di queste attività?

	Meno degli altri	Come gli altri	Più degli altri	Peggio degli altri	Come gli altri	Meglio degli altri
a.__________	☐	☐	☐	☐	☐	☐
b.__________	☐	☐	☐	☐	☐	☐
c.__________	☐	☐	☐	☐	☐	☐

DOPO ESSERTI ASSICURATO DI AVERE RISPOSTO A TUTTE LE DOMANDE VAI ALLA PAGINA SUCCESSIVA

Youth Self-Report for Ages 11-18 Copyright 2001 T.Achenbach, ASEBA,University of Vermont (USA)
Questionario sul Comportamento del Giovane (11-18 anni). Riproduzione autorizzata. Versione italiana curata da A. Frigerio (Istituto Scientifico "E.Medea" Ass. La Nostra Famiglia, Bosisio Parini (LC)). Traduzioni A. Frigerio (Istituto Scientifico "E.Medea" Ass. La Nostra Famiglia, Bosisio Parini (LC)); F. Giannotti e F. Cortesi (Università "La Sapienza" di Roma); A. Milone (Istituto Scientifico Stella Maris, Calambrone (Pi))© 20001

Riproduzioni non autorizzate sono perseguibili per legge

III. Per favore elenca qualsiasi organizzazione, club, squadra o gruppo a cui appartieni

☐ Nessuno

Rispetto agli altri ragazzi della tua età quanto sei attivo in ciascuna di queste?

	Meno attivo degli altri	Attivo come gli altri	Più attivo degli altri
a.______________________________	☐	☐	☐
b.______________________________	☐	☐	☐
c.______________________________	☐	☐	☐

IV. Per favore elenca qualsiasi lavoro o lavoretto che fai. Per esempio, fare il/la babysitter, rifare il letto, lavorare in un negozio, etc. (inclusi i lavori remunerati e non remunerati).

☐ Nessuno

Rispetto agli altri ragazzi della tua età come svolgi queste attività?

	Peggio degli altri	Come gli altri	Meglio degli altri
a.______________________________	☐	☐	☐
b.______________________________	☐	☐	☐
c.______________________________	☐	☐	☐

V. **1. Più o meno, quanti amici intimi hai? (non includere fratelli e sorelle)** ☐ **Nessuno** ☐ **1** ☐ **2 o 3** ☐ **4 o più**

2. Più o meno, quante volte alla settimana fai delle cose con gli amici al di fuori degli orari scolastici? (non includere fratelli e sorelle) ☐ **Meno di una** ☐ **1 o 2** ☐ **3 o più**

VI. Rispetto agli altri ragazzi della tua età, in che modo:

	Peggio degli altri	Come gli altri	Meglio degli altri	
a. Vai d'accordo con i tuoi fratelli e sorelle?	☐	☐	☐	☐ Io non ho fratelli o sorelle
b. Vai d'accordo con gli altri ragazzi?	☐	☐	☐	
c. Vai d'accordo con i tuoi genitori?	☐	☐	☐	
d. Fai delle cose da solo?	☐	☐	☐	

DOPO ESSERTI ASSICURATO DI AVERE RISPOSTO A TUTTE LE DOMANDE VAI ALLA PAGINA SUCCESSIVA

VII. Attuale rendimento scolastico ☐ **Non vado a scuola perché:** ________________________
____

Fai una crocetta per ogni voce

	Fallimento/ Insuccesso	Al di sotto della media	Intorno alla media	Sopra la media
a. Lettura, scrittura, grammatica o letteratura italiana	☐	☐	☐	☐
b. Storia o Studi Sociali	☐	☐	☐	☐
c. Aritmetica o Matematica	☐	☐	☐	☐
d. Scienze	☐	☐	☐	☐
Altre materie scolastiche per esempio: geografia, lingue straniere, informatica (non includere Educazione Fisica, o altre materie non scolastiche).				
e.______________________	☐	☐	☐	☐
f.______________________	☐	☐	☐	☐
g.______________________	☐	☐	☐	☐

Hai qualche malattia, disabilità o handicap? ☐ **No** ☐ **Si per favore descrivere**:

Per favore descrivi qualsiasi preoccupazione o problema che hai riguardo la scuola:

Per favore descrivi qualsiasi altra preoccupazione che hai:

Per favore descrivi le cose migliori che ti riguardano:

DOPO ESSERTI ASSICURATO DI AVERE RISPOSTO A TUTTE LE DOMANDE VAI ALLA PAGINA SUCCESSIVA

Di seguito è riportato un elenco di affermazioni che descrivono i ragazzi. Per ogni voce che ti descrive allo **stato attuale o negli ultimi sei mesi ,** sei pregato di fare una crocetta: sul due **(2)** se l'affermazione è **molto vera, o per lo più vera;** sull' uno **(1)** se l'affermazione è **in parte o qualche volta vera;** sullo zero **(0)** se l'affermazione **non è vera.**

0= Non vero **1= In parte o qualche volta vero** **2 = Molto vero o spesso vero**

**0**	**1**	**2**	1.	Agisco in modo infantile per la mia età
**0**	**1**	**2**	2.	Bevo alcolici senza l'approvazione dei miei genitori (descrivere): ___ ________________________
**0**	**1**	**2**	3.	Discuto in modo polemico
**0**	**1**	**2**	4.	Non porto a termine le cose che comincio
**0**	**1**	**2**	5.	Ci sono veramente poche cose che mi divertono.
**0**	**1**	**2**	6.	Amo gli animali
**0**	**1**	**2**	7.	Mi vanto
**0**	**1**	**2**	8.	Non riesco a concentrarmi o a mantenere l'attenzione a lungo
**0**	**1**	**2**	9.	Non riesco a evitare certi pensieri (descrivere): ______________ ________________________
**0**	**1**	**2**	10	Non riesco a stare seduto tranquillo
**0**	**1**	**2**	11	Dipendo troppo dagli adulti
**0**	**1**	**2**	12	Mi sento solo
**0**	**1**	**2**	13	Mi sento confuso o con la testa nel pallone
**0**	**1**	**2**	14	Piango molto
**0**	**1**	**2**	15	Sono molto onesto
**0**	**1**	**2**	16	Sono malvagio con gli altri
**0**	**1**	**2**	17	Sogno molto ad occhi aperti
**0**	**1**	**2**	18	Intenzionalmente mi faccio del male o ho tentato il suicidio
**0**	**1**	**2**	19	Cerco di ottenere molta attenzione
**0**	**1**	**2**	20	Distruggo le mie cose
**0**	**1**	**2**	21	Distruggo cose che appartengono agli altri
**0**	**1**	**2**	22	Disobbedisco ai miei genitori
**0**	**1**	**2**	23	Disobbedisco a scuola
**0**	**1**	**2**	24	Non mangio come dovrei
**0**	**1**	**2**	25	Non vado d'accordo con gli altri ragazzi
**0**	**1**	**2**	26	Non mi sento in colpa dopo aver fatto qualcosa che non avrei dovuto fare
**0**	**1**	**2**	27	Sono geloso degli altri
**0**	**1**	**2**	28	Infrango le regole a casa, a scuola, o altrove
**0**	**1**	**2**	29	Ho paura di certi animali, situazioni o posti al di fuori della scuola (descrivere):__________ ________________________
**0**	**1**	**2**	30	Ho paura di andare a scuola
**0**	**1**	**2**	31	Ho paura di poter pensare o fare qualcosa di male
**0**	**1**	**2**	32	Sento di dover essere perfetto
**0**	**1**	**2**	33	Sento che nessuno mi ama
**0**	**1**	**2**	34	Mi sento perseguitato dagli altri
**0**	**1**	**2**	35	Mi sento privo di valore o inferiore
**0**	**1**	**2**	36	Accidentalmente mi faccio spesso male
**0**	**1**	**2**	37	Sono coinvolto spesso in zuffe e liti
**0**	**1**	**2**	38	Vengo spesso preso in giro dagli altri
**0**	**1**	**2**	39	Frequento cattive compagnie
**0**	**1**	**2**	40	Sento suoni o voci che altre persone pensano non ci siano (descrivere):_________________ ______________________
**0**	**1**	**2**	41	Agisco senza fermarmi a pensare
**0**	**1**	**2**	42	Preferisco stare da solo piuttosto che con gli altri
**0**	**1**	**2**	43	Sono bugiardo o imbroglione
**0**	**1**	**2**	44	Mi mangio le unghie
**0**	**1**	**2**	45	Sono nervoso o teso
**0**	**1**	**2**	46	Movimenti nervosi o tic (descrivere): _______________ ________________________
**0**	**1**	**2**	47	Ho incubi
**0**	**1**	**2**	48	Non piaccio agli altri ragazzi
**0**	**1**	**2**	49	Posso fare certe cose meglio della maggior parte degli altri ragazzi
**0**	**1**	**2**	50	Sono troppo timoroso o ansioso
**0**	**1**	**2**	51	Soffro di vertigini o di stordimenti
**0**	**1**	**2**	52	Mi sento troppo colpevole
**0**	**1**	**2**	53	Mangio troppo
**0**	**1**	**2**	54	Mi sento esageratamente stanco senza una buona ragione

DOPO ESSERTI ASSICURATO DI AVERE RISPOSTO A TUTTE LE DOMANDE VAI ALLA PAGINA SUCCESSIVA

0= Non vero (per ciò che ne sai) 1= In parte o qualche volta vero 2 = Molto vero o spesso vero

**0**	**1**	**2**	55	Sono in soprappeso
			56	Problemi fisici **senza cause mediche conosciute**
**0**	**1**	**2**	a	Dolori (non includere mal di stomaco e mal di testa)
**0**	**1**	**2**	b	Mal di testa
**0**	**1**	**2**	c	Nausea, malessere
**0**	**1**	**2**	d	Problemi agli occhi (non includere se superati con lenti correttive) (descrivere): _______________ ________________________
**0**	**1**	**2**	e	Eruzione cutanea o altri problemi di pelle
**0**	**1**	**2**	f	Dolori di stomaco
**0**	**1**	**2**	g	Vomito, conati
**0**	**1**	**2**	h	Altro (descrivere):___________ ________________________
**0**	**1**	**2**	57	Assalgo fisicamente le persone
**0**	**1**	**2**	58	Mi stuzzico la pelle o altre parti del corpo (descrivere):________ ________________________
**0**	**1**	**2**	59	Posso essere molto amichevole
**0**	**1**	**2**	60	Mi piace provare cose nuove
**0**	**1**	**2**	61	Il mio rendimento scolastico è scarso
**0**	**1**	**2**	62	Sono poco coordinato o impacciato nei movimenti
**0**	**1**	**2**	63	Preferisco stare con ragazzi più grandi che con ragazzi della mia età
**0**	**1**	**2**	64	Preferisco stare con ragazzi più piccoli che con ragazzi della mia età
**0**	**1**	**2**	65	Mi rifiuto di parlare
**0**	**1**	**2**	66	Ripeto certe azioni di continuo (descrivere):________________ ________________________
**0**	**1**	**2**	67	Scappo via da casa
**0**	**1**	**2**	68	Strillo molto
**0**	**1**	**2**	69	Sono riservato, tengo le cose per me
**0**	**1**	**2**	70	Vedo cose che altre persone pensano non ci siano (descrivere): ___________________________ _______________________
**0**	**1**	**2**	71	Sono ipersensibile o mi imbarazzo facilmente
**0**	**1**	**2**	72	Appicco fuochi
**0**	**1**	**2**	73	Sono abile nei lavori manuali
**0**	**1**	**2**	74	Mi metto in mostra o faccio il pagliaccio
**0**	**1**	**2**	75	Sono troppo riservato o timido
**0**	**1**	**2**	76	Dormo di meno della maggior parte dei ragazzi
**0**	**1**	**2**	77	Dormo di più della maggior parte dei ragazzi durante il giorno e/o la notte(descrivere):___________ ________________________
**0**	**1**	**2**	78	Sono disattento o facilmente distraibile
**0**	**1**	**2**	79	Ho problemi nel parlare (descrivere): _______________ ________________________
**0**	**1**	**2**	80	Lotto per i miei diritti
**0**	**1**	**2**	81	Rubo in casa
**0**	**1**	**2**	82	Rubo fuori di casa
**0**	**1**	**2**	83	Accumulo moltissime cose che non mi servono (descrivere):________ ________________________
**0**	**1**	**2**	84	Faccio cose che altre persone pensano siano strane (descrivere): ___________________________ _______________________
**0**	**1**	**2**	85	Ho pensieri che altre persone riterrebbero strani (descrivere): __ ________________________
**0**	**1**	**2**	86	Sono testardo
**0**	**1**	**2**	87	Ho repentini cambiamenti di umore o di stati d'animo
**0**	**1**	**2**	88	Mi piace stare con altre persone
**0**	**1**	**2**	89	Sono sospettoso
**0**	**1**	**2**	90	Bestemmio o uso un linguaggio osceno
**0**	**1**	**2**	91	Penso di uccidermi
**0**	**1**	**2**	92	Mi piace far ridere gli altri
**0**	**1**	**2**	93	Parlo troppo
**0**	**1**	**2**	94	Prendo molto in giro gli altri
**0**	**1**	**2**	95	Sono irascibile
**0**	**1**	**2**	96	Penso al sesso eccessivamente
**0**	**1**	**2**	97	Minaccio la gente
**0**	**1**	**2**	98	Mi piace aiutare gli altri
**0**	**1**	**2**	99	Fumo, mastico o sniffo tabacco
**0**	**1**	**2**	100	Ho disturbi del sonno (descrivere): ________________________ ________________________
**0**	**1**	**2**	101	Marino la scuola o salto delle lezioni
**0**	**1**	**2**	102	Non ho molte energie

DOPO ESSERTI ASSICURATO DI AVERE RISPOSTO A TUTTE LE DOMANDE VAI ALLA PAGINA SUCCESSIVA

0= Non vero (per ciò che ne sai) **1= In parte o qualche volta vero** **2 = Molto vero o spesso vero**

**0**	**1**	**2**	103	Sono scontento, triste, o depresso
**0**	**1**	**2**	104	Sono più rumoroso degli altri ragazzi
**0**	**1**	**2**	105	Faccio uso di droga (**non** includere alcool o tabacco) (descrivere):___ __________________________
**0**	**1**	**2**	106	Mi piace essere corretto con gli altri
**0**	**1**	**2**	107	Mi piacciono gli scherzi
**0**	**1**	**2**	108	Mi piace prendere la vita così come viene
**0**	**1**	**2**	109	Cerco di aiutare gli altri quando posso
**0**	**1**	**2**	110	Desidero essere del sesso opposto
**0**	**1**	**2**	111	Chiuso in me stesso, non socializzo con gli altri
**0**	**1**	**2**	112	Mi preoccupo molto

Per favore scrivi qualsiasi altra cosa che descrive i tuoi sentimenti, comportamenti o interessi

ASSICURATI DI AVER RISPOSTO ATUTTE LE DOMANDE.

SOCIAL RESPONSIVENESS SCALE
QUESTIONARIO PER I GENITORI
John N. Constantino, M.D.

Nome del soggetto:________________________________ **Sesso: M F**

Data di nascita: ___/___/___ **Età:**_____ **Classe:**_______

Informazioni ottenute da: **Madre** **Padre** **Altro**__________ **Data di compilazione:**___/___/___

Istruzioni: Per piacere rispondete, per ogni affermazione, barrando la casella che meglio descrive il comportamento del bambino **negli ultimi sei mesi**..

	Non vero	Qualche volta vero	Spesso vero	Quasi sempre vero
1. Sembra più irrequieto in situazioni sociali rispetto a quando è solo	❑	❑	❑	❑
2. Le espressioni del suo volto non sono congrue con quello dice	❑	❑	❑	❑
3. Sembra sicuro di sè quando interagisce con gli altri	❑	❑	❑	❑
4. Quando è sotto stress il bambino sembra comportarsi in modo meccanico (per esempio, mostra comportamenti rigidi ed inflessibili che appaiono bizzarri)	❑	❑	❑	❑
5. Non si accorge quando gli altri provano ad "approfittarsi" di lui	❑	❑	❑	❑
6. Preferisce stare da solo piuttosto che con altri	❑	❑	❑	❑
7. Si rende conto di ciò che gli altri pensano o sentono	❑	❑	❑	❑
8. Si comporta in modo tale da sembrare strano o bizzarro	❑	❑	❑	❑
9. Deve stare sempre vicino agli adulti, sembra troppo dipendente da loro	❑	❑	❑	❑
10. Prende le cose troppo alla lettera e non coglie il reale significato della conversazione	❑	❑	❑	❑
11. E' abbastanza sicuro di se stesso	❑	❑	❑	❑
12. E' capace di comunicare i suoi sentimenti agli altri	❑	❑	❑	❑
13. Ha difficoltà nelle interazioni con gli altri che prevedono uno scambio di turni (per esempio, non sembra capire i turni in una conversazione)	❑	❑	❑	❑
14. Non è ben coordinato nell'attività fisica	❑	❑	❑	❑

	Non vero	Qualche volta vero	Spesso vero	Quasi sempre vero
15. E’ capace di capire gli stati d’animo delle altre persone dal tono di voce e dalle espressioni facciali	❑	❑	❑	❑
16. Evita il contatto oculare o ha un contatto oculare inusuale	❑	❑	❑	❑
17. Riconosce quando qualcuno si comporta in modo disonesto	❑	❑	❑	❑
18. Ha difficoltà a stringere amicizie anche quando ci mette tutto il suo impegno	❑	❑	❑	❑
19. Diviene frustrato quando non riesce a comunicare le sue idee	❑	❑	❑	❑
20. Mostra interessi sensoriali insoliti (come portare gli oggetti alla bocca o far ruotare gli oggetti) o strani modi di utilizzare i giocattoli	❑	❑	❑	❑
21. E’ capace di imitare le azioni degli altri	❑	❑	❑	❑
22. Gioca in modo appropriato con i bambini della sua età	❑	❑	❑	❑
23. Non partecipa ad attività di gruppo spontaneamente (a meno che non glielo si dica	❑	❑	❑	❑
24. Ha più difficoltà rispetto agli altri bambini nei cambiamenti della sua routine	❑	❑	❑	❑
25. Sembra non importargli/le di “stare al passo” o di non “essere sulla stessa lunghezza d’onda” degli altri	❑	❑	❑	❑
26. Offre conforto agli altri quando sono tristi	❑	❑	❑	❑
27. Evita di iniziare interazioni sociali con i coetanei o con gli adulti	❑	❑	❑	❑
28. Pensa o dice la stessa cosa più e più volte	❑	❑	❑	❑
29. E’ considerato dagli altri bambini strano o bizzarro	❑	❑	❑	❑
30. Si irrita in situazioni in cui accadono troppe cose (situazioni mo vimentate)	❑	❑	❑	❑
31. Non riesce a distogliere l’attenzione da una cosa una volta che ha iniziato a pensarci	❑	❑	❑	❑
32. Ha una buona igiene personale	❑	❑	❑	❑
33. E’ goffo nei rapporti sociali, anche quando cerca di essere cortese	❑	❑	❑	❑
34. Evita le persone che vogliono essergli emotivamente vicine	❑	❑	❑	❑
35. Ha difficoltà nel sostenere il flusso di una normale conversazione (scambi turni)	❑	❑	❑	❑
36. Ha difficoltà nel “relazionarsi” con adulti	❑	❑	❑	❑
37. Ha difficoltà nel “relazionarsi” con i coetanei	❑	❑	❑	❑
38. Risponde appropriatamente ai cambiamenti di umore negli altri (per es. quando l’umore di un amico o compagno di giochi cambia da felice a triste)	❑	❑	❑	❑
39. Ha una gamma d’interessi ristretta o insolitamente limitata	❑	❑	❑	❑

	Non vero	Qualche volta vero	Spesso vero	Quasi sempre vero
40. Ha una buona immaginazione, è bravo nel "far finta" (senza perdere il contatto con la realtà)	❑	❑	❑	❑
41. Passa da un'attività all'altra senza una finalità precisa	❑	❑	❑	❑
42. Si mostra esageratamente sensibile a suoni, stimoli tattili o odori	❑	❑	❑	❑
43. Si distacca facilmente dai genitori	❑	❑	❑	❑
44. Non capisce come un evento sia correlato ad un altro, allo stesso modo di come lo capiscono i suoi coetanei (per es. ha problemi nel capire i rapporti di causa ed effetto	❑	❑	❑	❑
45. Rivolge la sua attenzione verso dove gli altri stanno guardando o ascoltando	❑	❑	❑	❑
46. Mostra frequentemente espressioni facciali troppo serie	❑	❑	❑	❑
47. Ha comportamenti sciocchi o ride inappropriatamente	❑	❑	❑	❑
48. Possiede il senso dell'umorismo, capisce gli scherzi	❑	❑	❑	❑
49. E' estremamente abile in alcune attività, ma non altrettanto in molte altre	❑	❑	❑	❑
50. Mette in atto dei comportamenti ripetitivi o compulsivi (es. sfarfallio delle mani o dondolio)	❑	❑	❑	❑
51. Ha difficoltà a rispondere alle domande direttamente e finisce per girare intorno all'argomento	❑	❑	❑	❑
52. Si accorge quando parla a voce troppo alta o diventa troppo chiassoso	❑	❑	❑	❑
53. Parla con le persone con un tono di voce inusuale (parla come un robot o come se stesse "tenendo una conferenza")	❑	❑	❑	❑
54 Sembra trattare gli altri come se fossero degli oggetti	❑	❑	❑	❑
55. Si accorge quando si avvicina troppo ad una persona e invade il suo spazio personale	❑	❑	❑	❑
56. Si intromette tra due persone mentre parlano	❑	❑	❑	❑
57. Viene spesso preso in giro	❑	❑	❑	❑
58. Sembra essere maggiormente interessato dai particolari delle cose piuttosto che dalla "intera figura" (per es. se gli chiedete di descrivere cosa succede in una storia, il bambino/a può parlare solo del tipo di vestiti che i personaggi indossano)	❑	❑	❑	❑
59. E' molto diffidente	❑	❑	❑	❑
60. È emotivamente distaccato, non mostra le sue emozioni	❑	❑	❑	❑
61. È inflessibile, gli è difficile cambiare idea/opinione	❑	❑	❑	❑
62. Fornisce delle motivazioni insolite o illogiche per giustificare le sue azioni	❑	❑	❑	❑

	Non vero	Qualche volta vero	Spesso vero	Quasi sempre vero
63. Tocca gli altri in modo insolito (per esempio, il bambino può toccare qualcuno solo per avere un contatto e quindi andare via senza dire niente)	❑	❑	❑	❑
64. È troppo teso nei contesti sociali	❑	❑	❑	❑
65. Si incanta e guarda nel vuoto	❑	❑	❑	❑

Via Ospedale 119, 09124 Cagliari. Segreteria: 070 609 3415; Reparto: 070 609 3416 / 3417
Direzione: Tel. (+39) 070 609 3510 /3442/3509; Fax (+039) 070 652593 E-mail: azuddas@unica.it

CRS-R

CPRS-R:S VERSIONE PER GENITORI FORMA BREVE

C. Keith Conners
Adattamento italiano a cura di M. Nobile, B. Alberti e A. Zuddas

Nome del soggetto ______________________________ Sesso: M ☐ F ☐

Data di nascita ___ / ___ / ___ (giorno mese anno) Età _____ Classe _____ Nome del genitore ______________________ Data odierna ___ / ___ / ___ (giorno mese anno)

ISTRUZIONI. Di seguito viene riportata una serie di problemi comuni che bambini e adolescenti presentano. Rispondete a ogni affermazione a seconda del comportamento di vostro figlio nell'ultimo mese. Per ogni affermazione, chiedetevi "Quanto ha rappresentato un problema in quest'ultimo mese?" e fate un cerchietto attorno alla risposta che vi sembra più appropriata. Se quanto affermato non ha mai rappresentato un problema, o se lo è stato raramente o molto poco di frequente, fate un cerchietto attorno a 0. Se ha rappresentato un problema in misura notevole, o molto spesso o di frequente, fate un cerchietto attorno a 3. Fate un cerchietto attorno a 1 o 2 per le altre situazioni. Rispondete a tutte le affermazioni.

	**NON VERO** (mai, raramente)	**IN PARTE VERO** (ogni tanto)	**ABBASTANZA VERO** (spesso, di frequente)	**MOLTO VERO** (molto spesso, molto frequente)
**1.** È poco attento/a, si distrae facilmente	0	1	2	3
**2.** È arrabbiato/a e permaloso/a	0	1	2	3
**3.** Ha difficoltà a svolgere o a completare i compiti di casa	0	1	2	3
**4.** È sempre in movimento o si comporta come se andasse "a motore"	0	1	2	3
**5.** Ha un tempo di attenzione limitato	0	1	2	3
**6.** Discute in modo polemico con gli adulti	0	1	2	3
**7.** Giocherella con le mani o i piedi o si agita sulla sedia	0	1	2	3
**8.** Non riesce a portare a termine quanto gli/le viene assegnato	0	1	2	3
**9.** È difficile da controllare nei centri commerciali o quando si va a fare la spesa	0	1	2	3
**10.** È disordinato/a o disorganizzato/a a casa o a scuola	0	1	2	3
**11.** Perde la pazienza	0	1	2	3
**12.** Ha bisogno che qualcuno lo/la segua attentamente per terminare quanto gli/le viene assegnato	0	1	2	3
**13.** Si applica solo se qualcosa lo/la interessa veramente	0	1	2	3
**14.** Corre di qua e di là o si arrampica in situazioni in cui non dovrebbe	0	1	2	3
**15.** È distratto/a o la capacità di mantenere l'attenzione costituisce un problema	0	1	2	3
**16.** È irritabile	0	1	2	3
**17.** Evita, è riluttante o ha difficoltà a impegnarsi in compiti che comportano uno sforzo mentale continuato (come i compiti a scuola o a casa)	0	1	2	3
**18.** È irrequieto/a nel senso che si agita	0	1	2	3
**19.** Si distrae quando gli/le si impartiscono istruzioni su come fare qualcosa	0	1	2	3
**20.** Ha un atteggiamento di sfida nei confronti degli adulti o rifiuta di svolgere quanto richiesto	0	1	2	3
**21.** Ha difficoltà a concentrarsi in classe	0	1	2	3
**22.** Ha difficoltà ad aspettare in fila o ad attendere il proprio turno in giochi o attività di gruppo	0	1	2	3
**23.** Si allontana dal posto in classe o in altre situazioni in cui dovrebbe restare seduto/a	0	1	2	3
**24.** Intenzionalmente fa cose che infastidiscono gli altri	0	1	2	3
**25.** Non segue le istruzioni e non termina i compiti o i lavoretti assegnati o le incombenze sul posto di lavoro (non per atteggiamento oppositivo o incapacità di comprendere le istruzioni)	0	1	2	3
**26.** Ha difficoltà a giocare o ad impegnarsi in attività di gioco in modo tranquillo	0	1	2	3
**27.** Si sente facilmente frustrato/a nei suoi sforzi	0	1	2	3

Attenzione. *Questo modulo è stampato in azzurro ed è formato da più fogli incollati fra di loro: ogni altra versione è contraffatta, NON LA UTILIZZI. La fotocopia è illegale, danneggia la ricerca e non garantisce il Suo diritto a svolgere un test valido ed attendibile.*

GIUNTI O.S. Organizzazioni Speciali

© 1997, Multi-Health Systems Inc. Copyright internazionale valido in tutti i Paesi aderenti all'Unione di Berna, alla Convenzione Universale e a quelle bilaterali del diritto d'autore. Tutti i diritti sono riservati. Sono vietate la traduzione e la riproduzione dell'opera o di parti di essa, l'immagazzinamento dei dati e la loro trasmissione in qualsiasi forma e con qualsiasi mezzo, compresa fotocopia, copia fotostatica e memorizzazione elettronica, se non espressamente autorizzata da Multi-Health Systems Inc. Copyright per l'edizione italiana © 2007, Multi-Health Systems Inc. L'edizione italiana, adattata e standardizzata, è pubblicata da Giunti O.S. Organizzazioni Speciali su licenza di Multi-Health Systems Inc.

Modulo autoscoring
